# Findings on the Relationship Between Intestinal Microbiome and Vasculitis

**DOI:** 10.3389/fcimb.2022.908352

**Published:** 2022-06-27

**Authors:** Boyuan Sun, Xin He, Wen Zhang

**Affiliations:** ^1^ Department of Rheumatology, Peking Union Medical College Hospital, Chinese Academy of Medical Science & Peking Union Medical College, Beijing, China; ^2^ M.D. Program, Peking Union Medical College, Beijing, China; ^3^ Department of Rheumatology, Peking Union Medical College Hospital, Chinese Academy of Medical Science & Peking Union Medical College, National Clinical Research Center for Dermatologic and Immunologic Diseases, State Key Laboratory of Complex Severe and Rare Diseases, Beijing, China

**Keywords:** intestinal microbiome, antineutrophil cytoplasmic antibody-associated vasculitis, immunoglobulin A vasculitis, Behcet’s disease, Kawasaki disease, vasculitis

## Abstract

The microbiome has been implicated in small-, medium-, large-, and variable-vessel vasculitis. Dysbiosis can frequently be found in vasculitis patients with altered microbial diversity and abundance, compared with those with other diseases and healthy controls. Dominant bacteria discovered in different studies vary greatly, but in general, the intestinal microbiome in vasculitis patients tends to contain more pathogenic and less beneficial bacteria. Improvement or resolution of dysbiosis has been observed after treatment in a few longitudinal studies. In addition, some molecular changes in intestinal permeability and immune response have been found in animal models of vasculitis diseases.

## Introduction

The microbiome is defined as the collection of all microbes, including bacteria, fungi, viruses, and protozoa, as well as their genes, that naturally live on or inside our bodies and have established a symbiotic relationship with us over time. In particular, the intestinal microbiome is the microbe resident within the intestinal tract. Gut dysbiosis may drive some diseases including autoimmune disorders by affecting mucosal immune homeostasis and gut barrier integrity. The relationship between the microbiome and autoimmune diseases, such as systemic lupus erythematosus and rheumatoid arthritis, has been demonstrated and is becoming a novel therapeutic target (De Luca and Shoenfeld, 2019). Vasculitis is a type of autoimmune disease characterized by vascular inflammation and destruction as the main pathological change. The onset of vasculitis is affected by environmental triggers, wherein the changes in gut microbiota play an important role. Thanks to the development of sequencing techniques, culture-independent analysis of the microbiome is now possible and leading to rapid progression in microbiome study. However, 16S rRNA sequencing also limits the subjects analyzed among bacteria and ignores fungi or viruses because they do not contain 16S rRNA. In this review, we aim to demonstrate the recent findings on the relationships between intestinal microbiome and vasculitis in a clinical setting, as well as determine the potential mechanism in human or animal models. In this review, not all types of vasculitis in the 2012 Chapel Hill Nomenclature (Jennette, 2013) have been included because only studies on the intestinal microbiome in immunoglobulin A vasculitis, antineutrophil cytoplasmic antibody (ANCA)-associated vasculitis, Kawasaki disease, Behcet’s disease, and microbiome at other sites in giant cell arteritis and Takayasu’s arteritis have been reported to date.

## Small-Vessel Vasculitis

### Immunoglobulin A Vasculitis

Immunoglobulin A vasculitis (IgAV) is a subtype of small-vessel vasculitis characterized by IgA-dominant immune-complex deposition at vessel walls, leading to necrotizing inflammation of the vessel wall and vascular damage (Arnold et al., 2022). IgAV and IgA nephropathy (IgAN) have been believed to be twin extremities of the same disease (Pillebout, 2021). A lower diversity and richness of the microbiome were found in pediatric IgAV patients, showing an increased relative abundance of *Parabacteroides* and *Enterococcus* and a decreased relative abundance of *Dialister*, *Roseburia*, and *Parasutterella*. Furthermore, the IgA level was found to be negatively correlated with the genus *Bifidobacterium (* Wang et al., 2018). A lower abundance of *Bifidobacterium* and a lower ratio of *Bifidobacterium*/*Escherichia coli* were found in acute abdominal IgAV patients by Lan et al. They also reported that interleukin (IL)-17 and related cytokines may be promoted by dysregulated intestinal microbiome. This fact indicates that the activation of IL-17 induced inflammatory reaction. It also reveals the relationship of IL-17 activation with the pathogenesis of abdominal IgAV as well as the injury of the intestinal barrier (Lan et al., 2019). Wen et al. reported that the gut microbiome in IgAV patients contained decreased levels of *Coriobacteriaceae* and dominant levels of *Bacteroidaceae*; they further reported that the metabolism and biosynthesis of unsaturated fatty acids, especially arachidonic acid and its metabolites, may play a role in the biogenesis of IgAV (Wen et al., 2021). It was further demonstrated that gram-positive bacteria can be the main force driving gut microbiome changes (Cao et al., 2021). In a mouse model, antibiotic treatment could significantly prevent glomerular inflammation, IgA1 mesangial deposition, and development of proteinuria, proving the critical role of the intestinal microbiome in nephrotoxic IgA1 generation and IgAN development (Chemouny et al., 2019). In a B-cell activation factor (BAFF)-induced IgAN mouse model, intestinal commensal flora was essential for the generation of IgA. Commensal bacteria-reactive IgA antibodies were also found, indicating the involvement of gut microbiome in IgA nephropathy (McCarthy et al., 2011). The increase in intestinal permeability has been well expounded in IgAN (Floege and Feehally, 2016). Therefore, it was reasonable to find that the application of montmorillonite powder could effectively maintain normal function of the intestinal mucosal barrier and could be used to treat abdominal IgAV (Gao et al., 2018). Another clinical research demonstrated that alanyl-glutamine-enriched nutritional support could reduce the dosage of the intravenous glucocorticoids and weight loss but caused no difference in the duration of hospital stay and post-discharge recurrence (Xiong et al., 2019). Interestingly, proton pump inhibitors could also be a risk factor of IgAV with no dose-dependent effect (Lin et al., 2021).

Dysbiosis is also found in IgAN patients (**Table 1**). Further, an increased abundance of *Escherichia–Shigella*, *Hungatella*, and *Eggerthella* has been observed in IgAN patients (Hu et al., 2020). Among Malaysian IgAN patients, a decreased abundance of *Euryarchaeota* phylum and an increased abundance of *Fusobacteria* phylum have been reported compared with healthy controls (Sugurmar et al., 2021). Among Chinese IgAN patients, a decreased abundance of *Bifidobacterium* and *Blautia* spp. and an increased abundance of *Bacteroides* and *Escherichia–Shigella* have been observed compared with healthy controls (Zhong et al., 2020). Another previous study constructed a unique metabolic network of IgAN, linking the gut and blood, involving bilirubin, trimethoprim, stearamide, phenylalanine, cis-9,10-epoxystearic acid, and phosphatidylethanolamine 17:0 (Wu et al., 2021). Another study reported that host genetics may affect the intestinal microbiome supported by identifying nine genetic variants associated with susceptibility to IgAN and abundance changes in gut microbes (He et al., 2021a). There are even four ongoing clinical trials involving intestinal microbiome on ClinicalTrials. (https://clinicaltrials.gov/ct2/results?cond=IgA+Nephropathy&term=microbiome&cntry=&state=&city=&dist=).

**Table 1:** Summary of microbiome studies in IgAV.

Reference	Disease	Controls	Site	Technique	Study design	Major findings
Wang et al., 2018	85 children with IgAV	70 healthy children	Feces	16S rRNA sequencing (V1–V2 regions)	Cross-sectional	Lower microbial diversity and richness of microbiome; Increased relative abundance of *Parabacteroides* and *Enterococcus*; Decreased relative abundance of *Dialister*, *Roseburia*, and *Parasutterella*; IgA level is negatively related to the genus *Bifidobacterium*.
Lan et al., 2019	26 children with acute abdominal IgAV	16 healthy children	Feces	16S rRNA sequencing	Cross-sectional	Lower quantity of *Bifidobacterium*; Lower ratio of *Bifidobacterium*/*Escherichia coli*; IL-17 related inflammatory reaction and intestinal barrier injury participate in the pathogenesis of IgAV.
Wen et al., 2021	58 children with IgAV	28 healthy children	Feces	16S rRNA sequencing (V4–V5 regions)	Cross-sectional	Dominant *Bacteroidaceae* and decreased *Coriobacteriaceae* at family level; The biosynthesis and metabolism of unsaturated fatty acid, especially arachidonic acid, contribute to the biogenesis of IgAV.
Cao et al., 2021	10 children with IgAV	9 matched healthy children	Feces	Metagenomic analysis	Clinical trial	Gram-positive bacteria mainly drives the gut microbiome shifts of IgAV; Treatment-responsive features include *Weissella*, *Faecalibacterium prausnitzii*, and *Bifidobacterium pseudocatenulatum* and three genes named ABC.GLN1.A/S/P.
Hu et al., 2020	17 IgAN patients	18 matched HC	Feces	16S rRNA sequencing (V3–V4 regions)	Cross-sectional	The abundance of *Fusobacteria* increased while that of *Synergistetes* decreased at the phylum level in IgAN patients compared to controls; *Escherichia*-*shigella*, *Hungatella*, and *Eggerthella* are the significantly increased genera in the IgAN group; *Escherichia*–*shigella* is negatively correlated with the estimated glomerular filtration rate but positively associated with the urinary albumin-to-creatinine ratio.
Sugurmar et al., 2021	36 IgAN patients	12 matched HC	Feces	16S rRNA sequencing (V3–V4 regions)	Cross-sectional	An increased *Fusobacteria* phylum and a decreased *Euryarchaeota* phylum in IgAN patients; No difference in Operational Taxonomic Unit or alpha diversity.
Zhong et al., 2020	52 IgAN patients	25 HCs	Feces	16S rRNA sequencing (V3–V4 regions)	Cross-sectional	Higher level of *Bacteroides* and *Escherichia*–*shigella* while lower level of *Bifidobacterium* and *Blautia* spp. Higher proportions of *Escherichia*–*shigella* and lower proportions of *Bifidobacterium* spp. correlated with high urine red blood cell count and proteinuria levels.
Wu et al., 2021	15 IgAN patients	30 matched HC	Feces	16S rRNA sequencing (V3–V4 regions)	Cross-sectional	Higher relative abundances of *Streptococcus* and *Enterococcus* and lower relative abundances of *Bacteroidetes* and *Bacteroides;* 6 metabolism, including bilirubin, trimethoprim, stearamide, phenylalanine, cis-9,10-epoxystearic acid, and phosphatidylethanolamine 17:0, are identified that link the gut with blood.
Watanabe et al., 2017	48 IgAN patients	21 RT patients, 30 TH children	Tonsillar crypts	16S rRNA sequencing (V4 region)	Cross-sectional	*Prevotella* spp., *Fusobacterium* spp., *Sphingomonas* spp., and *Treponema* spp. predominant in IgAN; Different abundance percentages of *Prevotella* spp., *Haemophilus* spp., *Porphyromonas* spp., and *Treponema* spp. in IgAN compared to tonsillar hyperplasia.
He et al., 2021b	31 IgAN patients	30 controls	Oral cavity	16S rRNA sequencing (V3–V4 regions)	Cross-sectional	Decreased microbial diversity in IgAN; Enriched relative abundance of *Capnocytophaga* and *SR1_genera_incertae_sedis* while decreased that of 17 genera, such as *Rothia;* 12 genera able to discriminate IgAN from health, including *Capnocytophaga*, *Rothia*, and *Haemophilus*. 7 functional profiles were enriched in IgAN, including glycosphingolipid biosynthesis, oxidative phosphorylation, and N-glycine biosynthesis.
Khasnobish et al., 2021	43 IgAN patients	20 CT patients, 33 UC patients, 65 HC	Salivary	16S rRNA sequencing (V1–V2 regions)	Cohort study	The genus *Neisseria*, *Gemella*, *Prevotella* can be helpful to segregate IgAN from healthy and chronic tonsillitis; The genus *Haemophilus is* only significantly different between the IgAN and ulcerative colitis; The overall microbial population was similar between chronic tonsillitis and IgAN; Abundant *Firmicute* phylum while lower *Firmicutes/Proteobacteria* ratio in IgAN.

CT, chronic tonsillitis; HC, healthy controls; RT, recurrent tonsillitis; TH, tonsillar hyperplasia; UC, ulcerative colitis.

The microbiome in tonsillar crypts in IgAN patients presented a similar pattern to recurrent tonsillitis but different from tonsillar hyperplasia (Watanabe et al., 2017). The oral microbiome may also play a potential role in the pathogenesis of IgAN with seven predictive functional profiles, including glycosphingolipid biosynthesis, oxidative phosphorylation, and N-glycan biosynthesis (He et al., 2021b). The salivary microbiome can be a potential biomarker for IgA nephropathy, as proved in the Japanese population (Khasnobish et al., 2021).

The intestinal microbiome has been shown to differ between IgAV or IgAN patients and the healthy controls at a population and metabolism level at different positions. Furthermore, treatment for intestinal microbiome either directly or indirectly through gut permeability or diet had shown efficiency, which supports the potential pathogenic role of intestinal microbiome in IgAV.

### ANCA-Associated Vasculitis

Antineutrophil cytoplasmic antibody (ANCA)-associated vasculitis represents a cluster of small-vessel vasculitis including microscopic polyangiitis (MPA), granulomatosis with polyangiitis (GPA), and eosinophilic granulomatosis with polyangiitis (EGPA), which are all associated with ANCA, mainly causing arteriole damage and having a similar renal histology (e.g., focal necrotizing pauci-immune crescentic glomerulonephritis). Because ANCA-associated vasculitis (AAV) is characterized by mucosal inflammation including upper and lower respiratory tract inflammation, most studies have focused on nasal microbiome; one such review has been conducted by Dekkema et al. (2021), which reviewed nasal microbiome studies on AAV and concluded that nasal dysbiosis is a common phenomenon in active AAV, and immunosuppressant treatment can ameliorate nasal microbiome disorder. However, because of different study designs and population selection, the reported results of nasal microbiome differed between studies. Accordingly, it is hard to determine a causal relationship between nasal dysbiosis and AAV (Dekkema et al., 2021).

Kidney damage is another major clinical manifestation of AAV. A previous study in a mouse model of AAV demonstrated Th17 cells migrating from the lamina propria of the bowel wall to the kidney, leading to glomerulonephritis. Culturing these mice in a germ-free condition or with antibiotic treatment can significantly downregulate the Th17 cells and relieve kidney inflammation, while *Citrobacter rodentium* infection can expand Th17 cells and aggravate kidney damage in mice (Krebs et al., 2016). Some cross-sectional studies have proved that alterations of gut microbiota (including reduced SCFA-producing taxons) existed in AAV and were also linked to kidney injury (**Table 2**) by analyzing the fecal samples of patients using 16s rRNA sequencing (Najem et al., 2018; Yu et al., 2021; Yu et al., 2022). Najem et al. reported that the dysbiosis of intestinal flora was linked to higher Birmingham Vasculitis Activity Scores (BVASs), and gut microbiota in remissive AAV patients was similar to that in healthy controls, indicating that immunosuppressant and antibiotic use leads to recovery of gut microbiota (Najem et al., 2018). Yu et al. reported that MPA patients have increased abundance of *Actinomyces* and *Streptococcus* and reduced SCFA-producing taxons in the gut microbiome, which may be the basis of the pathogenesis of kidney damage in MPA (Yu et al., 2021). They also established a random forest model algorithm based on operational taxonomic unit (OUT) markers for the diagnosis and prediction of disease activity in MPA, which showed good predictive power (Yu et al., 2021). Another study also reported a gut–kidney axis in AAV, showing that changes in gut microbiota composition are associated with the degree of renal injury in AAV patients; this suggested that targeted regulation of gut dysbiosis may be a new intervention to reduce kidney damage in AAV patients (Yu et al., 2022).

**Table 2:** Summary of gut microbiome studies in AAV.

Reference	Disease	Controls	Site	Technique	Study design	Major findings
Najem et al., 2018.	29 active and 20 remission AAV	14 HC	Feces	16S rRNA sequencing (V1–V2 regions)	Cross-sectional	Reduced *Dialister, Prevotella, Faecalibacterium, Sutterella* in active AAV (vs. remissive AAV) Active AAV associated with gut dysbiosis Higher BVAS correlated with increased dysbiosis
Yu et al., 2021.	35 active and 36 remission MPA with kidney injury	34 HC	Feces	16S rRNA sequencing (V3–V4 regions)	Cross-sectional	Decreased α-diversity in MPA Increased *Actinomyces, Streptococcus* in MPA Reduced *Subdoligranulum, Eubacterium hallii, Ruminococcaceae UCG013, Eubacterium ventriosum, Dorea, Butyricicoccus* (all the 6 genera are SCFA-producing taxons)
Yu et al., 2022.	23 AAV with kidney injury	15 LN, 27 HC	Feces	16S rRNA sequencing (V3–V4 regions)	Cross-sectional	Decreased α-diversity in AAV Significant differences in α- and β-diversity among AAV, LN and HC Increased *Enterococcus, Peptoclostridium, Streptococcus, Akkermansia* in AAV Reduced *Faecalibacterium, Prevotella_9, Roseburia* in AAV *Deltaproteobacteria, unclassified_o_Bacteroidales, Prevotellaceae, Desulfovibrionaceae Paraprevotella, Lachnospiraceae_NK4A136_group* were correlated negatively with serum creatinine and positively with eGFR

AAV, antineutrophil cytoplasmic antibody-associated vasculitis; MPA, microscopic polyangiitis; BVAS, Birmingham Vasculitis Activity Score; HC, healthy controls; LN, lupus nephritis; SCFA, short-chain fatty acids.

In conclusion, besides the nasal microbiome, a certain correlation also exists between gut microbiota disturbance and the occurrence of AAV, especially in kidney injury. The mechanism can be summarized as follows: gut microbiota disturbance leads to reduced butyrate production and induction of Th17 differentiation; Th17 can then migrate to the kidney, mediating the development of renal injury. Immunosuppressant and antibiotic use can relieve kidney injury by recovering the gut microbiota, and this can be observed in the mouse model and in AAV patients. However, no clinical trials have been reported investigating whether a microbiota intervention therapy, such as dietary intervention, probiotics intake, or fecal transplant, can help with the treatment of AAV. Therefore, mechanism research and clinical application with regard to relationships between gut microbiota and AAV need further investigation.

## Medium-Vessel Vasculitis

### Kawasaki Disease

**Figure 1:**
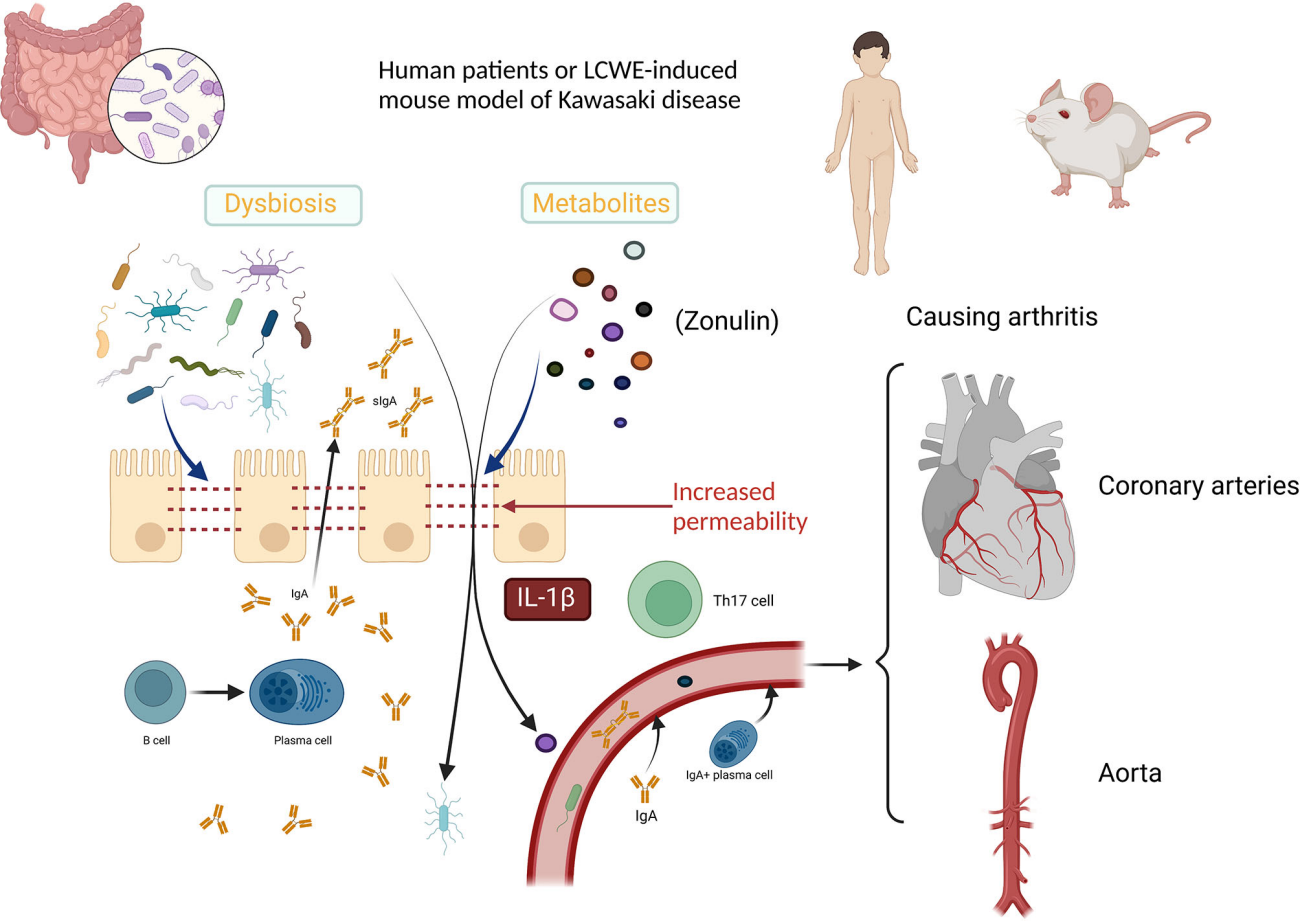
Potential mechanism between intestinal microbiome dysbiosis and Kawasaki disease. The increased permeability, probably caused by the immune response to dysbiosis, allows the microbiota and metabolites to get through the intestinal epithelial barrier. Abnormal inflammatory status involving increased production of IL-1β and Th17 contributes to the permeability increase. Microbiota, metabolites, and sIgA spread systemically along with the IgA+ plasma cell perforates into the vessel wall. IgA, immunoglobin A; IL-1β, interleukin-1β; LCWE, Lactobacillus-cell wall extract; sIgA, secretory Immunoglobulin A; Th17 cells, T helper 17 cells; ZO-1, zonula occludens-1. Created with BioRender.com.

Kawasaki disease (KD) is an acute febrile illness and systemic vasculitis that can cause coronary artery aneurysms (Noval Rivas and Arditi, 2020). A marked plasma cell response within the aorta with unusual IgA production was discovered in fatal acute KD, indicating a critical role of IgA in the pathogenesis of KD (Rowley et al., 1997). The intestinal microbiome affected by the environment was found to play a role in KD development (Esposito et al., 2019; Rhim et al., 2019). *Bacteroidetes* and *Dorea* are significantly associated with KD (Shen et al., 2020); *Fusobacteria*, *Shigella*, and *Streptococcus* also probably contribute to the onset of KD (Khan et al., 2020). The abundance of *Ruminococcus* is relatively increased during non-acute KD, whereas that of *Streptococcus* is relatively increased during acute KD (Kinumaki et al., 2015). A study comparing the microbiome in the throat, rectum, and venous blood detected similar cDNA sequences in both blood microbiome and intestinal microbiome (Abe et al., 2015). In a *Lactobacillus*-cell wall extract (LCWE) murine model, gut microflora was proven to play a critical role in the development of KD and both bacteria and fungi could control the induction and severity of KD (Wakita et al., 2015). Further, Th17 may be involved in the pathogenesis of acute KD (Yeung et al., 2015). Previously, the paracellular pathway opening function of Zonulin has been proved, which could increase intestinal permeability as a protective strategy (El Asmar et al., 2002). In the LCWE murine model, a relationship between intestinal permeability and KD was clarified, and the activation of the IL-1β signaling pathway, the elevated serum level of Zonulin, and the protective role of anti-Zonulin peptide in KD were reported (Rivas et al., 2017; Noval Rivas et al., 2019). Another study demonstrated that higher serum levels of zonula occludens-1 (ZO-1) were associated with failure of the first dose of intravenous immunoglobulin (IVIg) and lower serum levels of ZO-1 were associated with coronary artery lesions in KD patients (Lai et al., 2021). Further, the serum level of ZO-1 was revealed to be correlated with inflammation in cirrhotic patients, although it is not a marker of gut permeability (de Boer et al., 2008; Karthikeyan et al., 2018; Power et al., 2021). Intestinal permeability can be measured by many approaches, and several biomarkers, such as citrulline and lipopolysaccharide (LPS), have been found (Schoultz and Keita, 2020). More studies in this area are needed to clarify the changes in gut permeability in KD patients. Clinically, a correlation between KD development and previous antibiotic administration has been demonstrated (Fukazawa et al., 2020). However, after immunoglobulin/antibiotic therapy, absence or suppression of pathogenic bacteria was observed, whereas propagation of beneficial bacteria has been reported otherwise (Khan et al., 2020). Because KD and IgAV are probably similar as they share the same pathogenic participator, IgA, damage to intestinal permeability in both diseases has been reported; further, a treatment that targets gut permeability has shown efficiency even in IgAN, indicating an important role of gut permeability in the pathogenesis of vasculitis involving IgA. Thus, a hypothesis can be inferred that a shift of the microbiome may induce an increase in intestinal permeability as an immune response, leading to an opportunity for pathogens within the intestinal tract to cause abnormal immune reactions. A potential mechanism between intestinal microbiome dysbiosis and KD is illustrated in **Figure 1**.

Not only differences in gut microbiome but also damage to intestinal permeability have been reported. A protective switch in intestinal microbiome after immunoglobulin/antibiotic therapy but also a pathogenic role of previous antibiotic treatment have been observed, indicating a significant role of intestinal microbiome in the development of KD. However, the underlying mechanism needs further study to prove or refute the hypothesis.

## Large-Vessel Vasculitis

### Giant Cell Arteritis

Giant cell arteritis (GCA) is granulomatous systemic vasculitis involving both large and medium-sized arteries. IL-6, IL-12B, and HLA-DRB, as well as Th17, Th1, monocytes, CD4^+^ T cells, and Treg, have been found to be involved in the onset of GCA (Robinette et al., 2021). There has been no study on intestinal microbiome in GCA, but studies on microbiome in blood and aorta have shown differences, such as a tendency toward a decreased abundance of *Actinobacteria*, increased abundance of *Proteobacteria*, and minimal *Bacteroidetes*, in GCA patients compared with non-inflammatory thoracic aortic aneurysms. Further, GCA patients have been reported to have varying abundances of *Proteobacteria*, *Bifidobacterium*, *Parasutterella*, and *Granulicatella* compared with non-GCA temporal arteritis as well as increased abundance of *Rhodococcus*, an unidentified *Cytophagaceae* family member in blood samples, and a difference in microbiome between temporal artery and thoracic artery (Funchain et al., 2014; Getz et al., 2019a; Hoffman et al., 2019; Desbois et al., 2021).

### Takayasu’s Arteritis

Takayasu’s arteritis (TAK) is a rare vasculitis mainly damaging the aorta and its branches. Up to now, we cannot find any reports on gut dysbiosis in TAK. Most studies have focused on infections especially tuberculosis, as reviewed by Espinoza et al. (2018). Some studies have reported on blood and blood vessel microbiome in TAK. The fact that normal or diseased blood vessels are not sterile has been reported by Clifford et al. (Clifford and Hoffman, 2015). The arterial microbiome in inflamed arteries was different from that in non-inflamed arteries (Getz et al., 2019b). Desbois et al. studied blood samples of patients with TAK, GCA, and healthy donors by 16S rDNA sequencing and found that patients with TAK presented with an increased abundance of *Clostridia*, *Cytophagia*, and *Deltaproteobacteria* and a decreased abundance of *Bacilli* compared with healthy donors and showed higher levels of *Candidatus*, *Aquiluna*, and *Cloacibacterium* compared with GCA patients. To some extent, the changes in microbiome are related to the inflammatory and autoimmune processes, but it is hard to exclude the possibility that the presence of these changes is a consequence of chronic inflammation in TAK (Desbois et al., 2021). In conclusion, the function of the intestinal microbiome in the pathogenesis of TAK is unclear and needs further investigation.

## Variable-Vessel Vasculitis

### Behcet’s Disease

**Figure 2:**
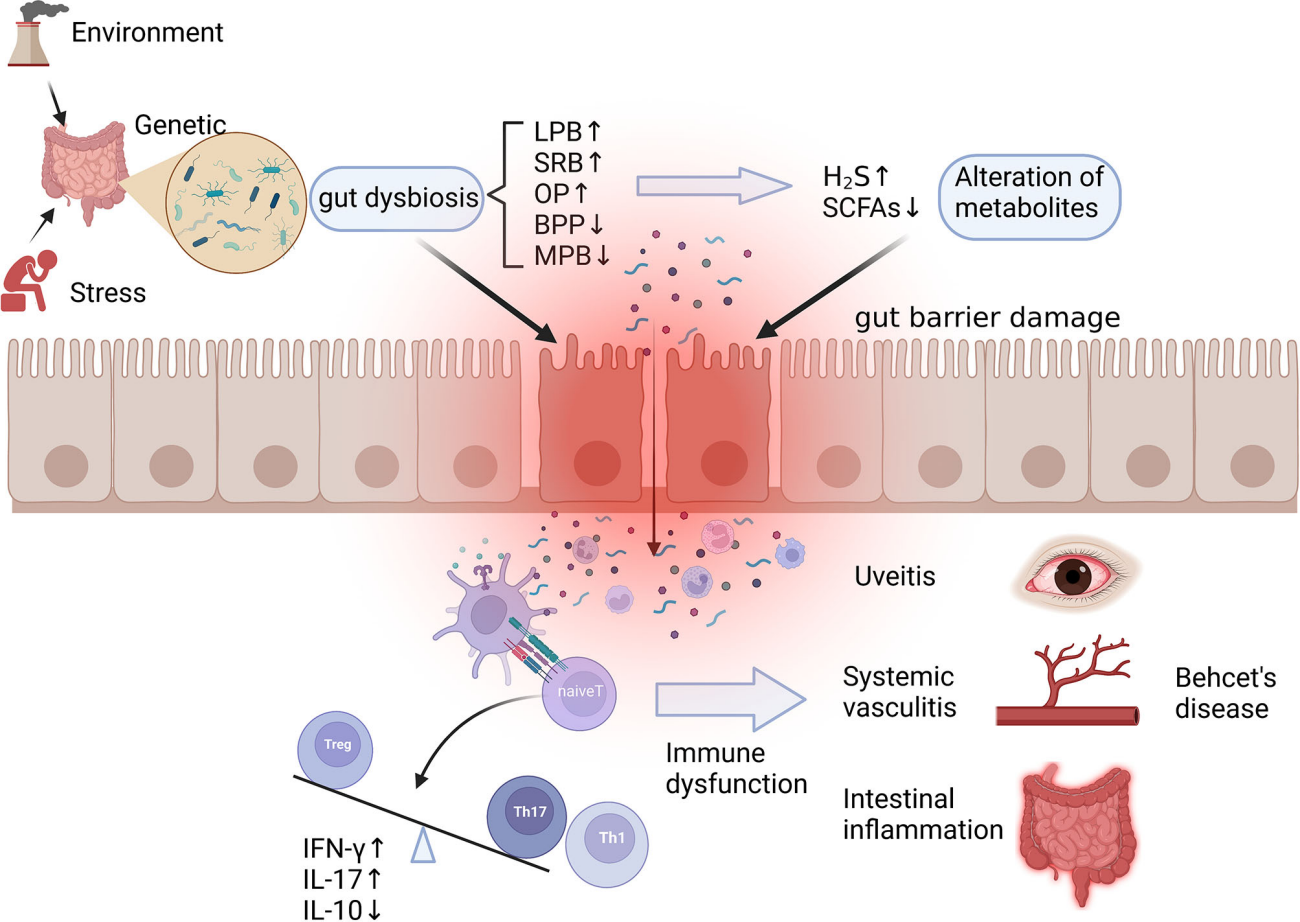
Potential mechanism of dysbiosis of gut microbiota in the pathogenesis of Behcet’s disease. Gut dysbiosis caused by environmental, stress, and genetic factors manifests as an increase in LPB, SRB, and OP and a decrease in BPB and MPB, resulting in alteration of microbial metabolites such as SCFAs and H2S. The above changes lead to the damage of the intestinal mucosal barrier, the invasion of microorganisms, and the leakage of their metabolites, which contribute to immune dysfunction such as overactivation of innate immune, increased differentiation of Th1 and Th17, and imbalance of Th17/Treg, ultimately helping the development of autoimmune reactions and systemic vasculitis. LPB, lactate-producing bacteria; SRB, sulfate-reducing bacteria; OP, opportunistic pathogen; BPB, butyrate-producing bacteria; MPB, methanogens; SCFAs, short chain fatty acids. Created with BioRender.com.

Behcet’s disease (BD) is a multisystem autoinflammatory disorder characterized by mucosal inflammation and systemic vascular lesions, presenting uveitis, recurrent oral aphthous ulcers, genital ulcers, and various vasculitis. Gut microbiota, as a host factor, has attracted wide attention in recent years because of its potential role in the pathogenesis of BD, but the relationship between intestinal flora and the occurrence of BD has not been clearly clarified. A potential mechanism between the gut dysbiosis and BD is described in **Figure 2**.

Some cross-sectional studies have reported that gut bacterial abundance changes in BD patients compared with healthy controls (**Table 3**) **(** Consolandi et al., 2015; Shimizu et al., 2016; Ye et al., 2018; Oezguen et al., 2019; Shimizu et al., 2019; Bilge et al., 2020; Tecer et al., 2020; van der Houwen et al., 2020; Kim et al., 2021). These results showed that the gut microbiota in BD patients was enriched with lactic acid-producing bacteria, sulfate-reducing bacteria, and some opportunistic pathogens but lacked butyric acid-producing bacteria and methanogenic bacteria. The results of gut microbiota changes in these studies are highly consistent, suggesting that changes in gut microbiota in BD patients are not influenced by region and ethnicity. An analysis of microbial functions by Ye et al. also revealed that the capsular polysaccharide transport system, oxidation–reduction process, and type III and type IV secretion systems were increased in active BD patients, indicating that abnormal gut microbiota of BD patients may be involved in BD development through these mechanisms (Ye et al., 2018). It should be noted that in almost all studies shown in **Table 2**, active BD patients receiving glucocorticoids or antibiotics treatment were excluded to avoid confounding factors. Therefore, it is hard to prove whether symptom improvement is partially achieved by medications for improving microbiota disorders. However, small intestinal bacterial overgrowth has been described in patients with gastrointestinal symptoms, and treatment of bacterial overgrowth by administration of antibiotics can improve symptoms (Jo et al., 2018). Whether these drugs have an impact on the structure of intestinal microbiota in patients with active BD needs further investigation.

**Table 3:** Summary of intestinal microbiome dysbiosis in Behcet’s disease patients compared to healthy controls.

Reference	Disease	Controls	Sites	Technique	Study design	Major findings
Consolandi et al., 2015	22 BD patients	16 co-habiting HCs, same diet and lifestyle	Feces	16S rRNA sequencing (V3–V4 regions)	Cross-sectional	Decreased α-diversity in BD; reduced *Roseburia, Subdoligranulum* in BD; Reduced butyrate and increased acetate in BD; *Roseburia* positively correlated with butyrate production
Shimizu et al., 2016	12 active BD patients	12 age- and sex-matched HCs	Feces	16S rRNA sequencing (V1–V2 regions)	Cross-sectional	No significant difference in α-diversity and OUT numbers; Significant difference in β-diversity in BD; Increased *Actinobacteria*, *Lactobacillus* species and decreased *Clostridia* in BD; Dysregulated SCFAs production in BD
Ye et al., 2018	32 active BD patients	74 sex-, age-, and BMI-matched HCs	Feces, oral cavity	16S rRNA sequencing (V3–V4 regions)	Cross-sectional	Increased sulfate-reducing bacteria *Bilophila* and several opportunistic pathogens *(Parabacteroides, Paraprevotella)* and decreased butyrate-producing bacteria *Clostridium* and methanogens *(Methanoculleus* and *Methanomethylophilus)* in BD feces; Enriched *Bifidobacterium, Prevotella*, and *Scar-dovia* in BD oral cavity
Oezguen et al., 2019	13 neuro-BD patients, 13 MS patients	14 HCs	Feces	16S rRNA sequencing (V3–V5 regions)	Cross-sectional	Significant differences in microbiota community composition. among BD, MS and HCs; Increased α-diversity in BD compared with HCs Increased *Parabacteroides, Clostridiales, Gemminger, Butyricimonas, Actinobacteria* and decreased *Vampirovibrio, Unclassified Lachnospiraceae* in BD Increases of different genera of the *Clostridiales* order drive dysbiosis in BD
Shimizu et al., 2019	13 BD patients	27 HCs	Feces	16S rRNA sequencing (V1–V2 regions)	Cross-sectional	No significant difference in α-diversity; Significant difference in β-diversity in BD; Increased *Eggerthella lenta, Acidaminococcus, Lactobacillus*, *Bifidobacterium bifidum, Streptococcus* and decreased *Megamonas hypermegale, Butyrivibrio*, *Streptococcus infantis, Filifactor* in BD; Decreased *Megamonas hypermegale* and *Butyrivibrio* species (SCFAs-producing bacteria) in BD; Decreased vitamin B syntheses pathway in BD gut microbiota
Bilge et al., 2020	27 BD patients	10 age-matched HCs	Feces	16S rRNA sequencing (V3–V4 regions)	Cross-sectional	No significant difference in α- and β-diversity; Increased *Actinomyces*, *Libanicoccus*, *Collinsella*, *Eggerthella*, *Enetrohabdus*, *Catenibacterium*, *Enterobacter* and decreased *Bacteroides, Cricetibacter, Alistipes, Lachnospira, Dielma, Akkermansia, Sutterella, Anaerofilum, Ruminococcease, Acetanaerobacterium, Copropaacter* in BD; Different clinical forms of BD have differences in gut microbiota composition.
Tecer et al., 2020	7 BD patients with uveitis	16 HCs, 12 FMF patients, 9 CD patients	Feces	16S rRNA sequencing (V2–4–8, V3–6, V7–9 regions); next-generation sequencing	Cross-sectional	Decreased α-diversity in BD compared with HC and FMF; Increased *Veionellaceae, Succinivibrionaceae, Succinivibrio, Mitsuokella* in BD*;* Fecal microbiota consisted mainly of *Firmicutes* as a phylum, *Clostridia* as a class, *Clostridiales* as an order and *Prevotella copri* as a species in BD; *Succinivibrionaceae* is dominant and the signature family, whereas *Bacteroides* was absent in BD patients.
van der Houwen et al., 2020	19 BD patients from the Netherlands, 13 from Italy, 18 from Dutch	17 HCs from the Netherlands, 15 from Italy, 15 from Dutch	Feces, oral cavity	16S rRNA sequencing (V3–V4 regions), fecal IgA-SEQ analysis	Cross-sectional	No significant differences in α-diversity between BD and HCs; Italian cohort displayed a significant lower α-diversity compared to the Dutch cohort; Increased *Bifidobacterium*, *Dorea*, *Ruminococcus bromei* and Decreased unclassified *Barnesiellaceae, Lachnospira* genera in BD; Oral Dutch BD microbiome displayed increased *Spirochaetaceae* and *Dethiosulfovibrionaceae* families.
Kim et al., 2021	9 BD patients, 7 RAU patients	9 BD-matched HCs, and 7 RAU-matched HCs	Feces, oral cavity	16S rRNA sequencing (V3–V4 regions)	Cross-sectional	No significant differences in α-diversity between BD and HCs; α-diversity decrease in BD with disease activity Active BD patients had more *Bacteroides uniformis* than their matched HCs and patients with the disease in an inactive state. BD patients had higher salivary *Rothia mucilaginosa* than RAUs patients BD patients with uveitis had different abundances of various taxa compared to those without uveitis.

HC, healthy control; BD, Behcet’s disease; MS, multiple sclerosis; FMF, familial Mediterranean fever; CD, Crohn’s disease; RAU, recurrent aphthous ulcer.

In mouse models, some studies have proved the relation between gut microbiota and BD. Wang et al. reported that the transplantation of fecal samples collected from active BD patients to experimental autoimmune uveitis (EAU) mice could exacerbate EAU activity and decrease the concentration of three kinds of SCFAs (Wang et al., 2021). In mice receiving BD feces, it was observed that the intestinal barrier was disrupted with low expression of tight-junction proteins, leading to LPS release into circulation. An enhancement of Th1 and Th17 cell differentiation in mesenteric lymph nodes and spleen and activation of neutrophils were also detected. Compared with the healthy control-recipient group, the BD-recipient group showed an increased mRNA expression of IFN-γ, IL-17, monocyte chemotactic protein-1 (MCP-1), IL-1β, and tumor necrosis factor-α (TNF-α); in contrast, IL-10 mRNA expression was decreased in the BD-recipient group compared with the control group. It was indicated that the gut microbiome can regulate the autoimmune response by increasing intestinal permeability and enhancing immunity. Otherwise, in accordance with the metagenomic sequencing results in the human gut microbiome, enrichment of *Bilophila*, *Alistipes*, and *Paraprevotella* was observed in the BD-recipient group. Islam et al. demonstrated that the environment and stress (especially noise stress) can affect the incidence of herpes simplex virus type 1 (HSV-1)-induced BD in mice. HSV-inoculated mice, under conventional conditions, reported a higher incidence of BD compared with mice under specific pathogen-free (SPF) conditions. More OTUs and bacterial phyla were observed in SPF mice, indicating that a higher microbial diversity can inhibit BD development (Islam et al., 2021a). Another study by Islam et al. reported higher *Tenericutes* and lower *Deferribacteres* and *Verrucomicrobia* in HSV-1-induced BD mice than in normal mice by 16s rRNA sequencing. In addition, they found that butyrate treatment can improve symptoms in BD mice by enhancing dendritic cells and Treg cells. Furthermore, a similar effect was observed by the administration of *Eubacterium rectale* (a kind of butyrate-producing bacteria) in BD mice, which can upregulate NK1.1+ cells and reduce serum IL-17 levels and disease severity scores, sharing the partial mechanism of colchicine, which is used to treat BD patients. These findings indicate that the use of *E. rectale* can be a potential treatment approach for BD patients (Islam et al., 2021b).

Besides bacterial preparations, diet may be a potential method to influence the gut microbiota and help relieve the severity of active BD phases. Two clinical trials (Pagliai et al., 2020; Emmi et al., 2021) have been conducted studying dietary intervention to change the illness state of BD patients. A proof-of-concept randomized trial recruited 17 BD patients and divided them into two groups with different butyrate-enriched diets for 3 months. Both diets improved patient condition by regulating blood redox status and promoting fibrin degradation, which are impaired in BD *via* reactive oxygen species (ROS). However, the 3-month dietary intervention did not influence gut microbiota composition and SCFA production, which may be attributed to the resistance stability of the gut microbial ecosystem. Therefore, long-term randomized controlled trials are needed to be conducted to further investigate the benefits of butyrate-enriched diet among BD patients. Another randomized, open, crossover clinical trial, named MAMBA, is still ongoing, where 90 patients with BD have been recruited and divided into three groups: a lacto-ovo-vegetarian diet group, a Mediterranean diet without supplement group, and a Mediterranean diet supplemented with butyrate group. BD severity was recorded during the follow-up period in this study, and the primary outcomes are changes in BD gastrointestinal and systemic symptoms compared with baseline. Secondary outcomes are changes in gut microbiota composition, SCFA production, inflammatory profile, and antioxidant profile. The results of this trial have not yet been published.

In brief, studies about BD are relatively more abundant compared with other vasculitis types. The imbalance of gut microbiota in BD patients has been verified by multiple 16S rRNA sequencing. The imbalance of gut microbiota leads to the dysfunction of the intestinal immune system, including the activation of innate immunity and the imbalance of adaptive immunity, causing the occurrence of BD. These mechanisms have been initially demonstrated in mouse models. In addition, clinical trials concerning microbial therapy have been conducted, and preliminary results have been obtained, but given the lack of time for dietary intervention, it is uncertain whether dietary intervention can be used for treating BD. Moreover, no relevant research has been performed on probiotic treatment, fecal transplantation, and other methods, which are worthy for further investigation.

## Conclusion

The intestinal microbiome has been explored in several types of vasculitis and dysbiosis, and decreased microbial diversity has been discovered in most conditions. The core point is that bacterial perturbations and metabolite changes can alter innate and adaptive immunity, thus being involved in the occurrence of vasculitis. There are similarities between some vasculitis types. For example, studies concerning IgAV, AAV, and BD all have reported that reduced unsaturated fatty acid or SCFA production is associated with intestinal immune inflammation. Impairment of intestinal barrier capacity caused by gut dysbiosis is involved in IgAV, KD, and BD. Further, Th17 and IL-17 may play an important role in IgAV, BD, and kidney injury in AAV. The uniqueness of each vasculitis type may differ from specific gut microbiota changes and differences in affected immune molecules and cells.

Although a relationship between the intestinal microbiome and treatment has been reported, studies that link the intestinal microbiome and the development of vasculitis as well as the treatment and diet sequentially are lacking. There are nearly no studies determining whether abnormality of gut microbiota can affect the drug use and treatment effect among patients. Studies on neoadjuvant treatments for vasculitis such as diet, probiotics, or fecal transplant have not yet been conducted or are still in animal experiment stage. We need more studies in basic biology and translational medicine to discover the microbial and molecular pathogenesis of vasculitis. There is potential to find some novel therapeutic targets and predict the efficiency of individualized treatment.

## Author Contributions

WZ designed this concept. BS collected and analyzed the literature and drafted the manuscript of IgAV, GCA, and KD. XH collected and analyzed the literature and drafted the manuscript of AAV, TAK, and BD. WZ edited the manuscript and revised the language. All authors contributed to the article and approved the submitted version.

## Funding

This work was supported by the National Natural Science Foundation of China (82071839) and CAMS Innovation Fund for Medical Sciences (CIFMS 2021-1-I2M-003).

## Conflict of Interest

The authors declare that the research was conducted in the absence of any commercial or financial relationships that could be construed as a potential conflict of interest.

The reviewer LF declared a shared affiliation with the authors BS, XH and WZ to the handling editor at the time of review.

## Publisher’s Note

All claims expressed in this article are solely those of the authors and do not necessarily represent those of their affiliated organizations, or those of the publisher, the editors and the reviewers. Any product that may be evaluated in this article, or claim that may be made by its manufacturer, is not guaranteed or endorsed by the publisher.
